# Root-Inspired Bio-Interlocking Structure Design and Its Mechanism on Enhancing the Interfacial Bonding of NiTi/Ti6Al4V Fabricated by MM-LPBF

**DOI:** 10.3390/ma19163516

**Published:** 2026-08-19

**Authors:** Jingyu Xu, Honglei Ge, Zhenyu Niu, Jiakun Shi, Shuitao Zhou, Juzhao Chen, Xuehao Gao, Haida Chen, Fenggang Liu

**Affiliations:** 1 Research Centre for Laser Extreme Manufacturing, Ningbo Institute of Materials Technology and Engineering, Chinese Academy of Sciences, Ningbo 315201, China; 2 Faculty of Mechanical Engineering and Mechanics, Ningbo University, Ningbo 315211, China; 3 National Defence Key Discipline Laboratory of Light Alloy Processing Science and Technology, Nanchang Hangkong University, Nanchang 330063, China

**Keywords:** laser powder bed fusion, root-inspired bio-structure, dissimilar metal joining, interfacial interlocking structure

## Abstract

The dissimilar combination of NiTi shape memory alloy and Ti6Al4V titanium alloy offers superelasticity, biocompatibility and high specific strength, showing broad application prospects in aerospace and medical fields. However, when fabricating NiTi/Ti6Al4V composite components by multi-material laser powder bed fusion (MM-LPBF), brittle cracks or even complete delamination easily occur at the interface. In this paper, without relying on intermediate interlayer materials, we innovatively propose a root-inspired three-dimensional bio-interlocking interface structure. By means of macroscopic three-dimensional geometric interlocking, the crack propagation path and load transfer mode are forced to change. Using the branching angle (45°, 60°) and the structural size multiplier (1.2, 1.5) as variables, the influence of the bio-inspired geometric parameters on the interfacial forming quality, microstructure and mechanical properties was systematically investigated. The results show that the branching angle is the primary factor determining the performance. The 45° low-angle branched specimens exhibit overall brittle delamination along the flat metallurgical reaction interface under shear loading, with an average shear strength of only 17.47 MPa. In contrast, the 60° high-angle branched specimens, owing to their larger normal embedding depth, exhibit a failure mode transitioning to a mixed mode that includes crack deflection, branch shearing and plastic tearing of the Ti6Al4V matrix. Although TEM confirms that a continuous Ti_2_Ni brittle phase still exists at the interface, the optimised 60–1.5 structure increases the average shear strength to 128.37 MPa, which is more than six times higher than that of the 45–1.2 group (17.47 MPa). This “geometrical constraint toughening” strategy provides a new paradigm for the interfacial strengthening of dissimilar metals without relying on metallurgical modification.

## 1. Introduction

Multi-material laser powder bed fusion (MM-LPBF) enables the integration of advantageous properties of different materials into a single component, attracting wide attention in aerospace and biomedical fields [1,2,3,4]. The combination of NiTi shape memory alloy and Ti6Al4V titanium alloy, taking advantage of superelasticity and high specific strength, can be used for key components such as implants (e.g., artificial joints, vascular stents) and aerospace adaptive structures (e.g., smart skins, morphing wings) [5,6]. However, when fabricating NiTi/Ti6Al4V by MM-LPBF, cracks or even complete delamination easily occur at the interface, severely limiting the engineering application of this technology [7]. Specifically: the melting points (NiTi approximately 1310 °C, Ti6Al4V approximately 1600 °C) and coefficients of thermal expansion (NiTi approximately 6.6–11.0 × 10^−6^/K, Ti6Al4V approximately 8.9–9.5 × 10^−6^/K) differ significantly; under the extremely high cooling rate, a continuous brittle Ti_2_Ni intermetallic compound tends to form at the interface; at the same time, residual stresses accumulate continuously during the layer-by-layer stacking process of MM-LPBF. The combined effect of these three factors causes interfacial cracking. In recent years, researchers have made important progress in the densification and phase transformation control of NiTi alloys by optimising the processing parameters [8,9,10].

To address the interfacial cracking issue, existing studies mainly focus on two strategies. The first involves the addition of intermediate interlayer materials to suppress the precipitation of brittle phases by improving metallurgical compatibility. For example, Oliveira et al. used a Cu interlayer for laser welding of NiTi/Ti6Al4V, and when the Cu layer thickness was optimised to 75 μm, the joint tensile strength reached 300 MPa, though brittle phases such as Ti_2_Cu still formed at the interface [11]. Zhang et al. employed a Nb interlayer and obtained a shear strength of about 90 MPa at a laser power of 400 W and scanning speed of 800 mm/s; the key mechanism was that Nb blocked the mutual diffusion of Ni and Ti, inducing a gradient transition zone consisting of β-Ti(Nb) solid solution and ductile Ti_2_Nb phases [12]. Moreover, Teshome et al. attempted a Pd interlayer and also observed a suppression effect on brittle phases [13]. Nevertheless, these methods all rely on a third-phase material, involve complex powder spreading and interfacial metallurgical control, and have stringent requirements on the heat input window.

The second strategy is to construct a mechanical joint at the interface, i.e., to achieve physical anchoring through geometric interlocking structures, redistributing the stress from a single intermetallic layer into the bulk matrix. In the fields of dissimilar material welding and multi-material additive manufacturing, mechanical interlocking at the interface has made significant progress. Chueh et al. designed an “interdigitated” interlocking structure for a 316L stainless steel/polymer interface, where metal interlocking skeletons were embedded into the polymer, significantly improving the bond strength [14]. Tang et al. successfully fabricated CoCrMo/Cu gradient components using a toothed interlocking interface via LPBF, and the interfacial shear strength was effectively enhanced [15]. Xu et al., by means of oscillating laser directed energy deposition, induced a unique interlocking microstructure at the SiCp/Al composite interface, increasing the tensile strength by 35% and the ductility by 14.5% [16]. For the refractory dissimilar metal system W-Cu, Guan et al. laser-machined high-aspect-ratio grooves on the tungsten surface to induce surface nano-texturing, successfully achieving W-Cu interlocking bonding; the tensile fracture strength approached that of diffusion bonding [17]. However, most of the above works were conducted on material systems with similar melting points or good metallurgical compatibility, such as polymer/metal, CoCrMo/Cu, and W-Cu. There are very few studies on mechanical interlocking for the interfacial brittleness problem of MM-LPBF fabricated NiTi/Ti6Al4V. The synergistic toughening mechanism between the brittle Ti_2_Ni phase (formed under the high cooling rate of LPBF) and the mechanical interlocking structure in NiTi/Ti6Al4V remains unclear, and thus requires urgent exploration. Beyond interlayer-based approaches, broader surface architecture engineering strategies have been reviewed by Sharma et al., who summarised advanced methods for improving interface properties through microstructural design and surface texturing in metal matrix composites [18]. Their review provides a wider context for the present geometrical interface optimisation strategy, suggesting that the combination of topological design with microstructural engineering could represent a future direction for further enhancing dissimilar metal interfaces. The concept of geometry-driven performance enhancement finds parallels in other manufacturing domains. Bukvić et al. demonstrated that geometric and material modifications in worm gears can simultaneously improve mechanical efficiency and durability [19], suggesting that structural optimisation—rather than purely compositional adjustment—can yield comparable performance gains. This perspective aligns closely with the present strategy of using macroscopic geometric interlocking to overcome interfacial brittleness.

In this study, we propose a root-inspired bio-interlocking structure as a disruptive preparation technique—overcoming the interfacial brittleness of dissimilar metals through macroscopic geometric structure design rather than material modification. Using the branching angle (45°, 60°) and the structural size multiplier (1.2, 1.5) as variables, four groups of bio-interlocking specimens were designed and prepared, and the NiTi/Ti6Al4V interface was integrally formed by MM-LPBF. Various other bio-inspired interface architectures, such as tree-, tooth-, hook-, and nacre-inspired designs, have also been explored in additive manufacturing for improving interfacial integrity. Compared with these configurations, the present root-inspired design offers distinct advantages for MM-LPBF of dissimilar metals: its three-dimensional branching network provides multi-axial constraint that is particularly effective against the isotropic nature of interfacial brittle phases, while its hierarchical geometry can be readily parameterised and scaled. The interfacial forming quality, microstructure and mechanical properties were systematically studied, and the geometric constraint toughening mechanism of the root-inspired bio-structure in the presence of metallurgical brittleness was revealed.

## 2. Experimental Materials and Methods

### 2.1. Materials

**Figure 1:**
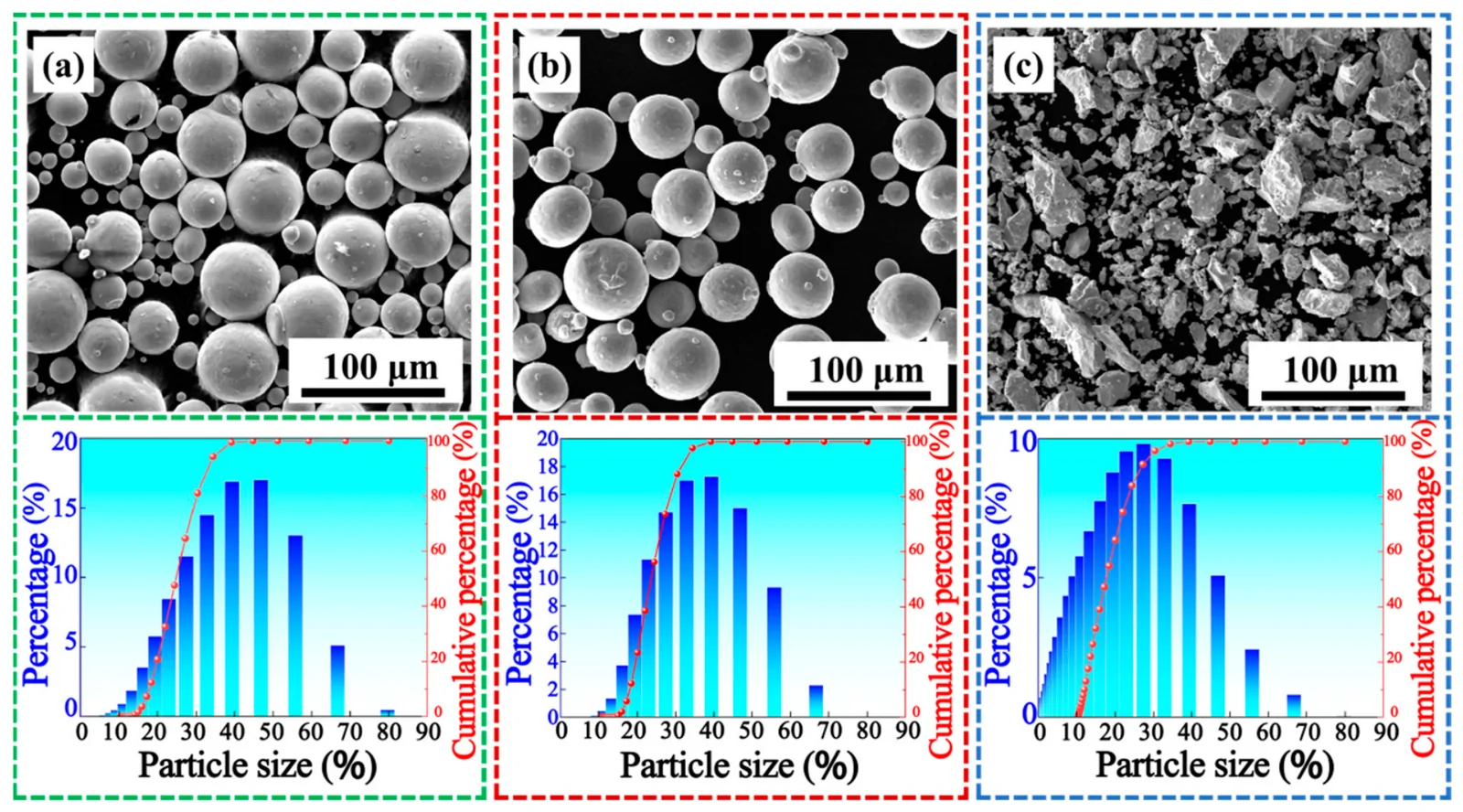
Powder morphology and particle size distribution: (**a**) NiTi; (**b**) Ti6Al4V; (**c**) Nb.

Spherical NiTi powder (Ni 54.5 wt.%; Avimetal Powder Metallurgy Technology Co., Ltd., Beijing, China) and Ti6Al4V powder (Liaoning Guanda New Material Technology Co., Ltd., Anshan, China) with a particle size of 15–53 μm and average diameters Dv(50) of 37.31 μm and 34.83 μm, respectively, were used. The morphologies and particle size distributions of the powders are shown in Figure 1, and their chemical compositions are listed in Table 1. The substrate was a φ60 mm forged NiTi/Ti6Al4V block.

### 2.2. Fabrication Process

A self-developed ultra-precision multi-material LPBF system equipped with a 500 W fibre laser (wavelength 1064 nm, focused spot size ~68 μm) was employed for sample fabrication. Multi-material composite samples were fabricated by laser powder bed fusion. By constructing a root-inspired bio-interlocking interface structure with three-dimensional interlocking features, the stress distribution in the NiTi/Ti6Al4V dissimilar material joint can be regulated and the bonding strength can be enhanced. In the experiments, four groups of root-supporting models with specific geometric characteristics were first designed using three-dimensional modelling software, with the branching angle and the diameter at the branch tip as the core variables. During the fabrication process, the Ti6Al4V alloy substrate was first printed on a Ti6Al4V base plate. When the printing reached the NiTi alloy root structures, the powder suction nozzle of the multi-material additive manufacturing equipment removed the excess Ti6Al4V powder from the current printed layer and subsequently filled the vacancies in the Ti6Al4V substrate with NiTi powder. Processing parameters for NiTi alloy: laser power 160 W, scanning speed 600 mm/s, layer thickness 30 μm, hatch spacing 80 μm. Processing parameters for Ti6Al4V alloy: laser power 300 W, scanning speed 600 mm/s, layer thickness 50 μm, hatch spacing 60 μm. A layer-wise alternating scanning strategy with a rotation of 67° was adopted to balance the thermal stresses. The oxygen content was kept below 65 ppm during the whole process.

### 2.3. Bio-Inspired Design

**Figure 2:**
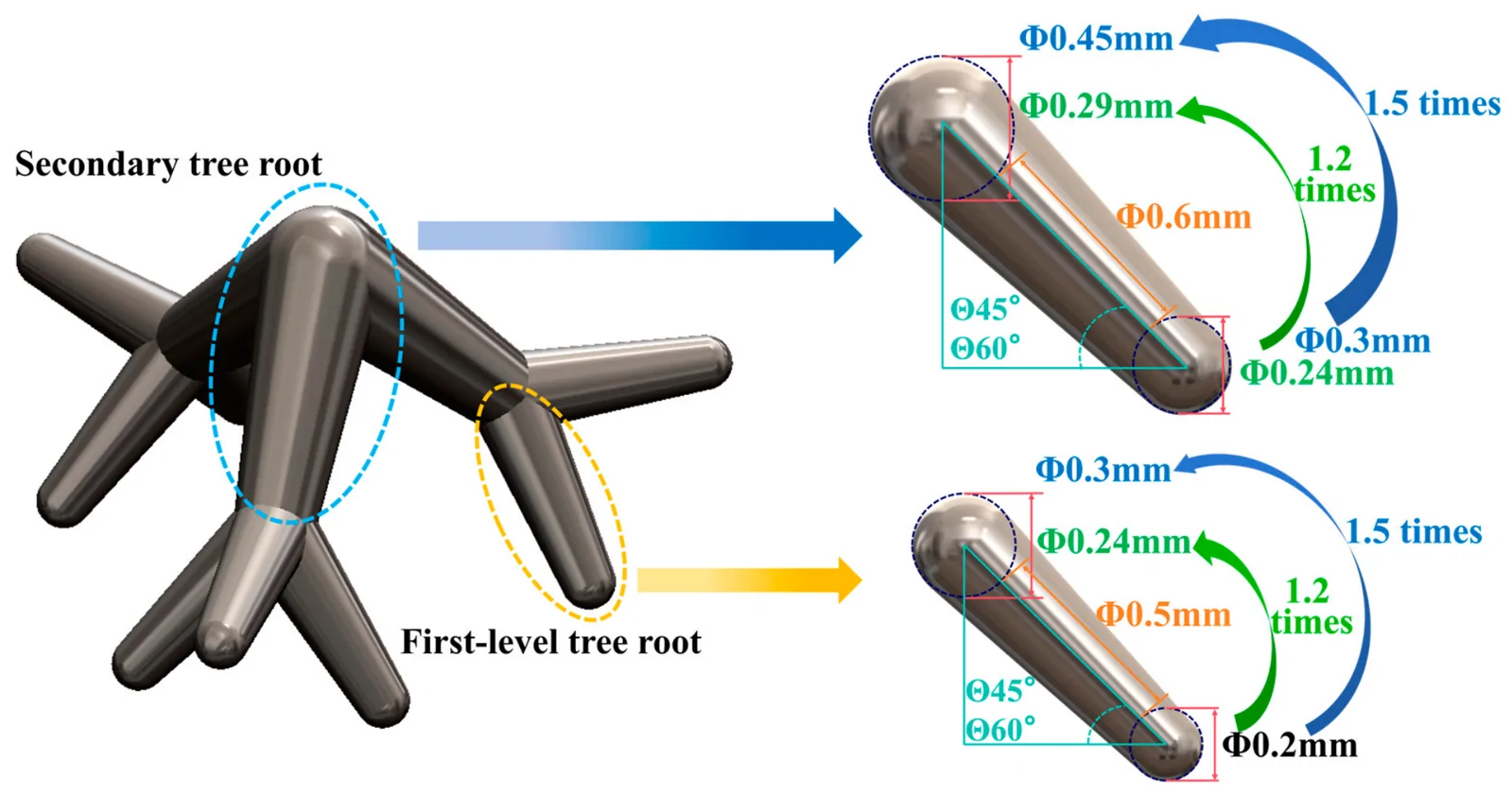
Design model and geometric parameters of the root-inspired bio-structure.

The root-inspired bio-structure is based on three-dimensional interlocking, as shown in Figure 2. The structure comprises a primary trunk and secondary branches. The sphere diameter at the starting end of the primary root is 0.2 mm, with a length of 0.5 mm; it increases stepwise along the axis by factors of 1.2 and 1.5. The sphere diameter of the first secondary branch is the same as that of the second node of the primary root, and also evolves by the same multipliers; the branch length is set to 1.2 times that of the primary root (0.6 mm). With branching angles θ of 45° and 60°, a 2 × 2 full-factorial experiment matrix (A1–A4, Table 2) was constructed. All shear strength data are reported as mean ± standard deviation (SD).

The selection of the structural dimensions (primary root length of 0.5 mm, branch length of 0.6 mm, sphere diameter of 0.2 mm, and size multipliers of 1.2 and 1.5) was guided by the following considerations. First, the minimum feature size of 0.2 mm was chosen to be compatible with the LPBF resolution limit (laser spot size ~68 μm) while ensuring reliable powder packing in the root cavities during multi-material powder deposition. Second, the branching angles of 45° and 60° were selected to represent two distinct mechanical regimes; 45° approximates the critical angle below which the normal embedding depth is insufficient to activate significant crack deflection, while 60° provides sufficient normal constraint to force crack deviation into the matrix. Third, the size multipliers of 1.2 and 1.5 were chosen to systematically investigate the effect of geometric scale on load transfer efficiency without exceeding the practical limits of powder flowability and laser penetration during fabrication.

**Figure 3:**
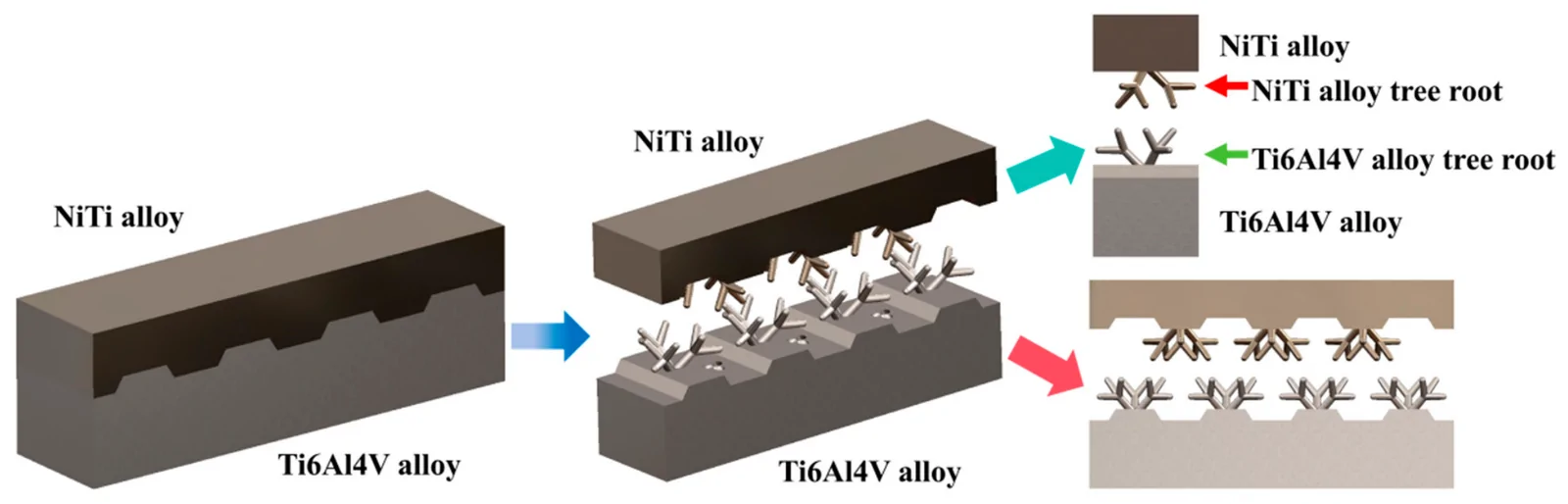
Schematic of the double-sided embedded rectangular bio-interlocking structure.

Figure 3 is a schematic of the rectangular bio-interlocking specimen, showing the double-sided riveting mode in which NiTi roots are embedded into the Ti6Al4V matrix and Ti6Al4V roots are embedded into the NiTi matrix.

**Figure 4:**
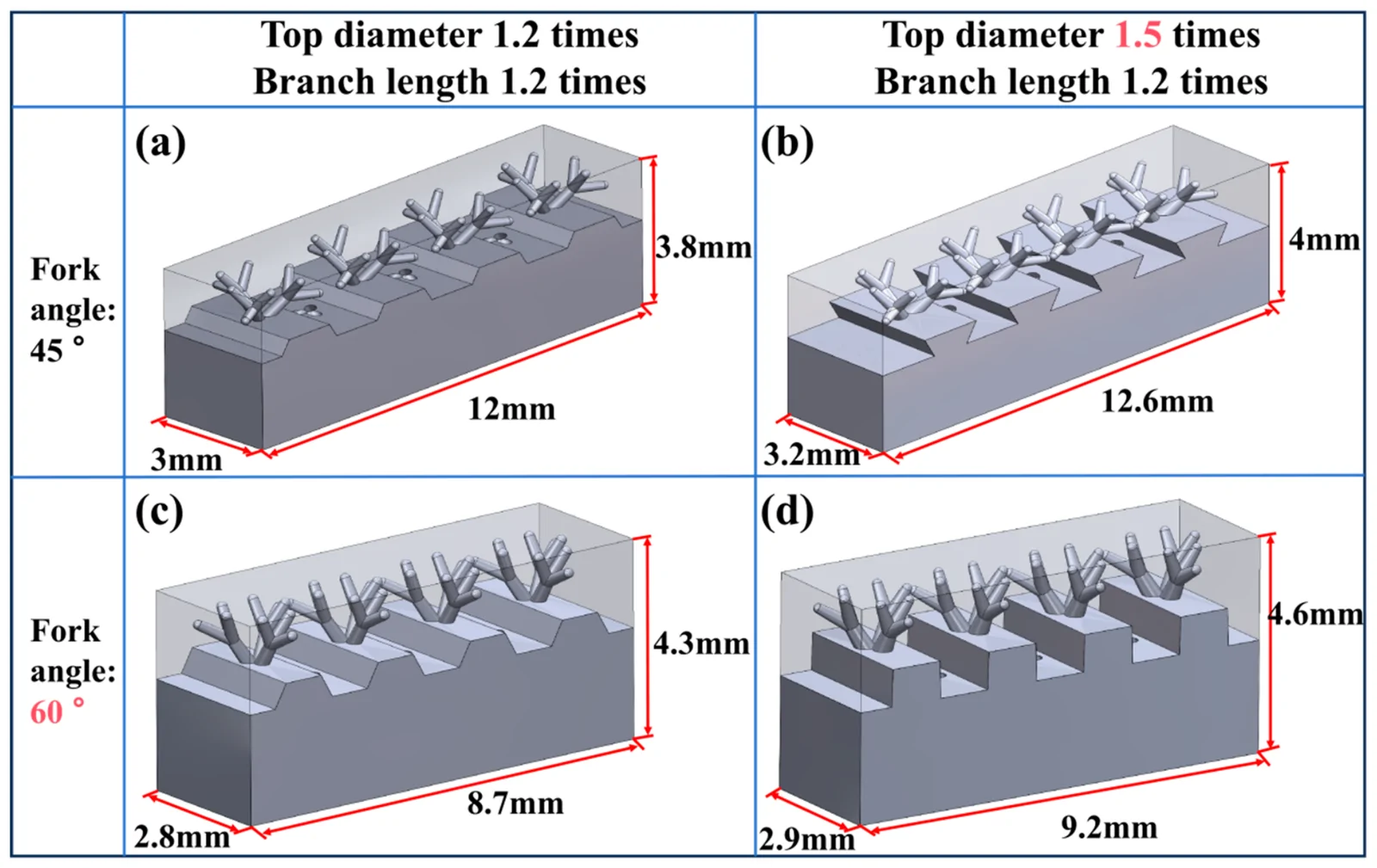
Three-dimensional modelling and parameter planning of the rectangular bio-interlocking specimens: (**a**) 45–1.2 group; (**b**) 45–1.5 group; (**c**) 60–1.2 group; (**d**) 60–1.5 group.

To accommodate the spatial distribution of the root structure, Figure 4 presents the three-dimensional modelling and parameter planning for the four specimen groups: the total length of the 45° branching group was set to 12–12.6 mm; because the branches of the 60° branching group are more vertically concentrated, the total length was optimised to 8.7–9.2 mm.

**Figure 5:**
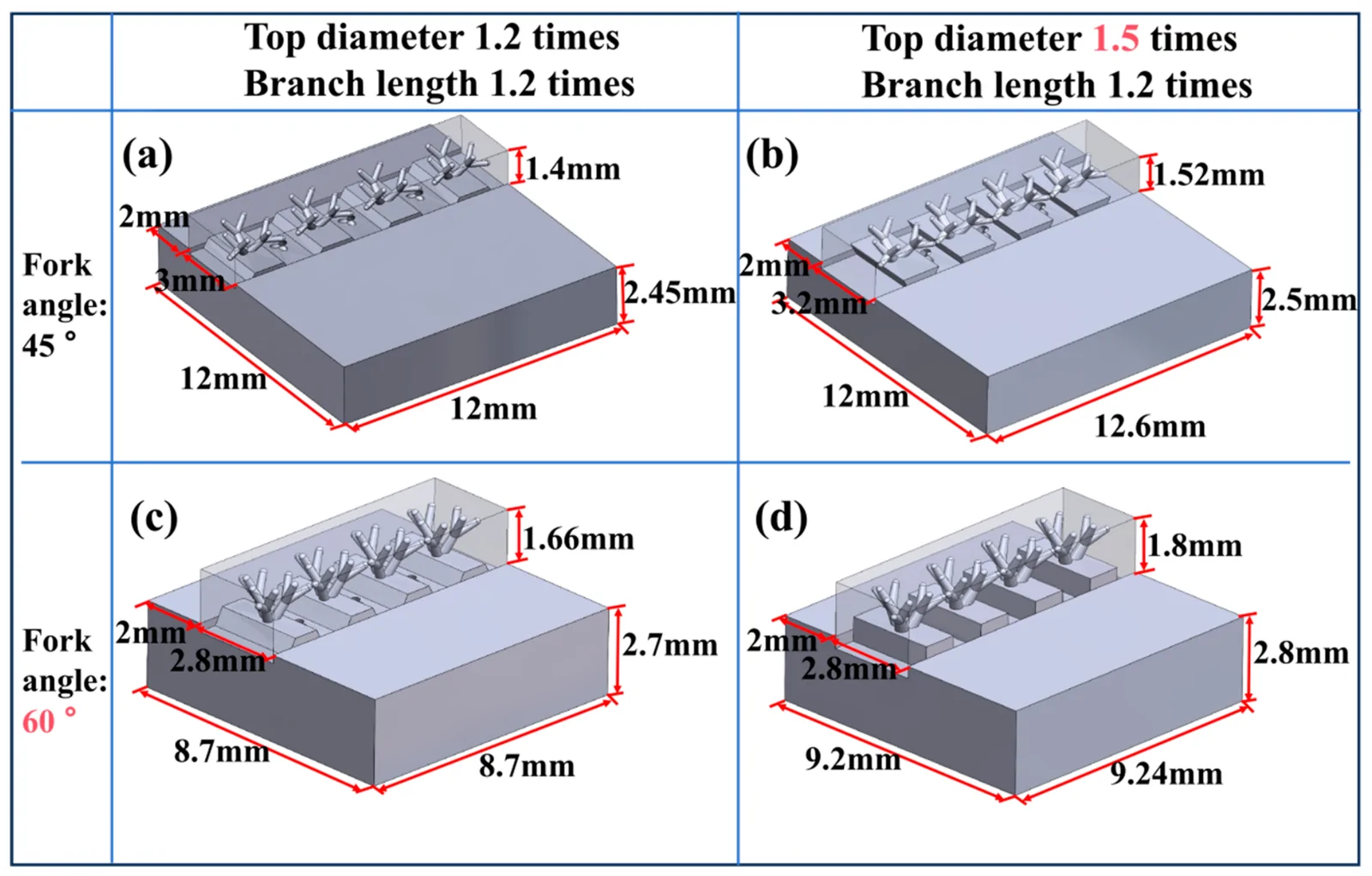
Schematic of shear-compression test specimens with different parameters: (**a**) 45–1.2 group; (**b**) 45–1.5 group; (**c**) 60–1.2 group; (**d**) 60–1.5 group.

Figure 5 shows the schematic of the shear-compression test specimens with different design parameters, visually illustrating how the branching angle and size multiplier affect the embedding depth.

### 2.4. Characterisation and Testing

A digital microscope (KEYENCE VHX-5000, KEYENCE, Osaka, Japan) and a scanning electron microscope (SEM; FEI QUANTA FEG 250, FEI, Hillsboro, OR, USA, and ZEISS SIGMA 300, ZEISS, Oberkochen, Germany) were used to observe the interfacial morphology. Energy-dispersive X-ray spectroscopy (EDS) was performed for elemental mapping and line scans. A focused ion beam (FIB; ZEISS Auriga, ZEISS, Oberkochen, Germany) was used to prepare thin slices for transmission electron microscopy (TEM). TEM observations (TALOS F200X, Thermo Fisher Scientific, Waltham, MA, USA), including selected area electron diffraction (SAED) and high-resolution imaging under STEM mode, were conducted to identify the phases. Compressive shear testing was carried out on a Zwick/Roell Z030 universal testing machine (ZwickRoell, Ulm, Germany) at a loading rate of 5 mm/min; at least three parallel samples per group were tested and the average values were taken.

## 3. Results and Discussion

### 3.1. Interfacial Defect Analysis

**Figure 6:**
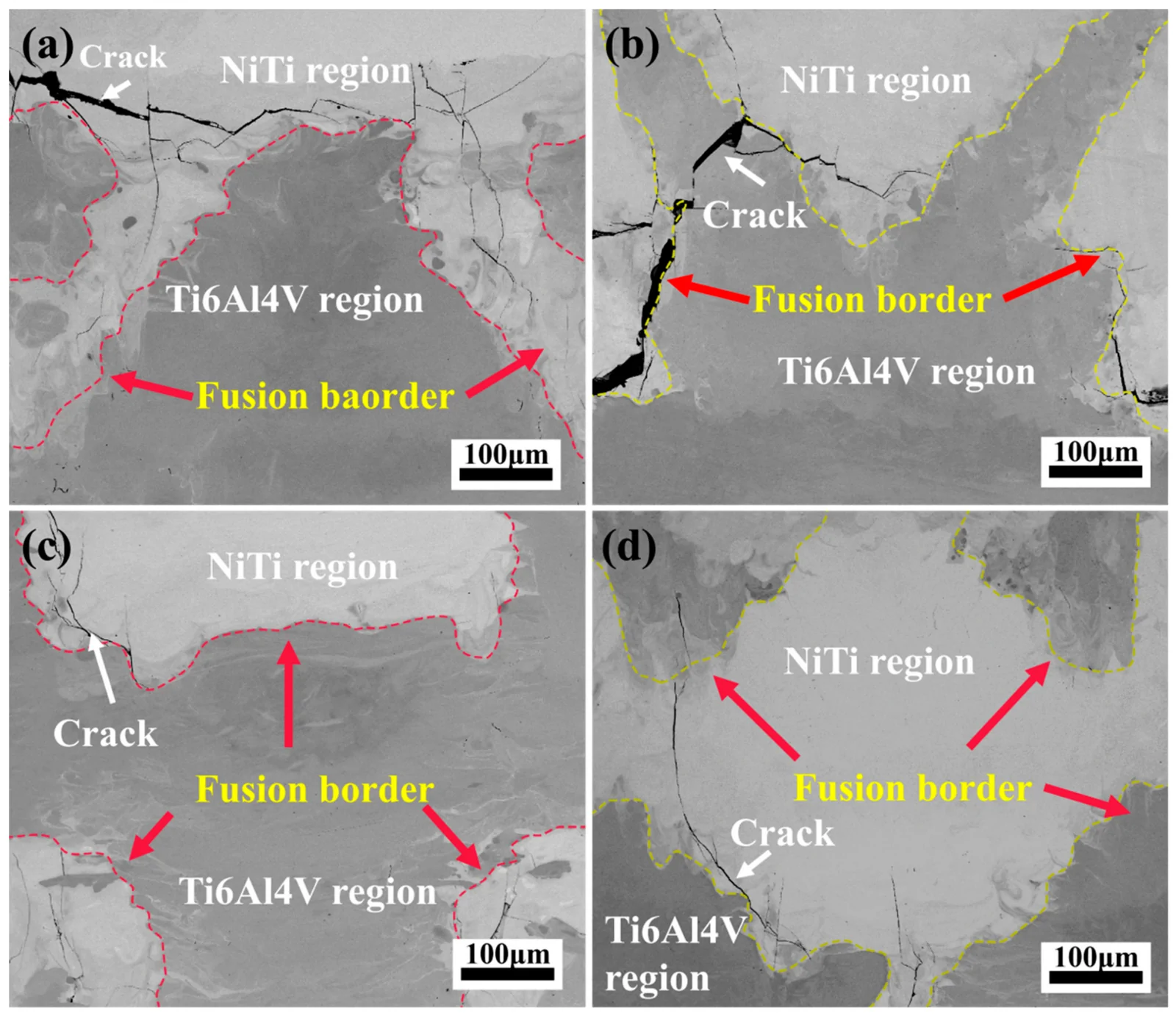
Comparison of interfacial SEM morphologies of the root-inspired bio-structures with different parameters: (**a**) 45–1.2 group; (**b**) 45–1.5 group; (**c**) 60–1.2 group; (**d**) 60–1.5 group.

Figure 6 presents a comparison of the interfacial SEM morphologies of the root-inspired bio-structures with different parameters. For the 45° branching group (Figure 6a,b), the fusion boundary exhibits a wavy shape with large undulations. In the 1.2-multiplier group (a), obvious penetrating cracks exist at the interface. In the 1.5-multiplier group (b), the crack path is deflected at the thickened branches. For the 60° branching group (Figure 6c,d), the interfacial continuity is significantly improved: in the 1.2-multiplier group (c), cracks are concentrated at the bottom corners of the trapezoidal shape and are narrow; in the 1.5-multiplier group (d), the fusion boundary is clear and continuous, with only local micro-cracks and no large-scale delamination. This indicates that a larger branching angle and a moderately increased size multiplier are beneficial for improving interfacial integrity.

To quantitatively evaluate the interfacial damage under different parameters, an automated image processing algorithm based on OpenCV was employed to statistically measure the crack area fraction from the SEM images in Figure 6. The results reveal that the 45° branched specimens exhibit considerably higher crack densities, with area fractions of 0.63% and 1.31% for the 1.2× and 1.5× groups, respectively. In contrast, the 60° branched specimens show significantly reduced cracking, with area fractions of only 0.22% and 0.26% for the 1.2× and 1.5× groups, respectively. These quantitative measurements confirm that a larger branching angle effectively suppresses crack formation and propagation, which is fully consistent with the morphological observations. It is worth noting that within the 45° group, increasing the size multiplier from 1.2 to 1.5 paradoxically increases the crack area fraction, suggesting that excessive geometrical dimensions may introduce additional thermal stress concentrations during the layer-by-layer stacking process of MM-LPBF, thereby promoting crack initiation.

To further quantitatively characterise the crack propagation trajectory, the projected crack length (Lp), actual crack path length (La), crack tortuosity (T = La/Lp), and maximum crack deflection angle (θd, max) relative to the nominal interface were measured from the SEM images using ImageJ software (version 1.54u8, National Institutes of Health, Bethesda, MD, USA). The results are summarised in Table 3. For the 45° branched groups (A1, A2), the tortuosity values are 1.12 and 1.22, with maximum deflection angles of approximately 20° and 30°, respectively, indicating relatively flat crack propagation with only moderate deviations. For the 60–1.2 group (A3), although its tortuosity (1.10) and deflection angle (20–25°) are comparable to those of the 45° groups, its significantly higher shear strength (122.61 MPa) suggests that the increased normal embedding depth effectively constrains crack opening, thereby preventing complete interfacial debonding even without extreme crack tortuosity. Notably, the highest-strength 60–1.5 group (A4) exhibits the largest tortuosity (T = 1.28) and the most substantial deflection angle (45–55°), confirming that the bio-interlocking geometry successfully forces the crack to deviate significantly from the flat metallurgical reaction layer, consuming substantially more fracture energy. These quantitative measurements are fully consistent with the observed shear strength trend and provide direct evidence supporting the proposed geometry-induced toughening mechanism.

The above quantitative analysis of crack morphology and propagation trajectory reveals a clear geometry-dependent cracking behaviour. To elucidate the underlying mechanisms, the elemental diffusion and phase constitution at the bio-inspired interface were further investigated by EDS, EPMA and TEM, as presented in the following sections.

### 3.2. Interfacial Composition Analysis

**Figure 7:**
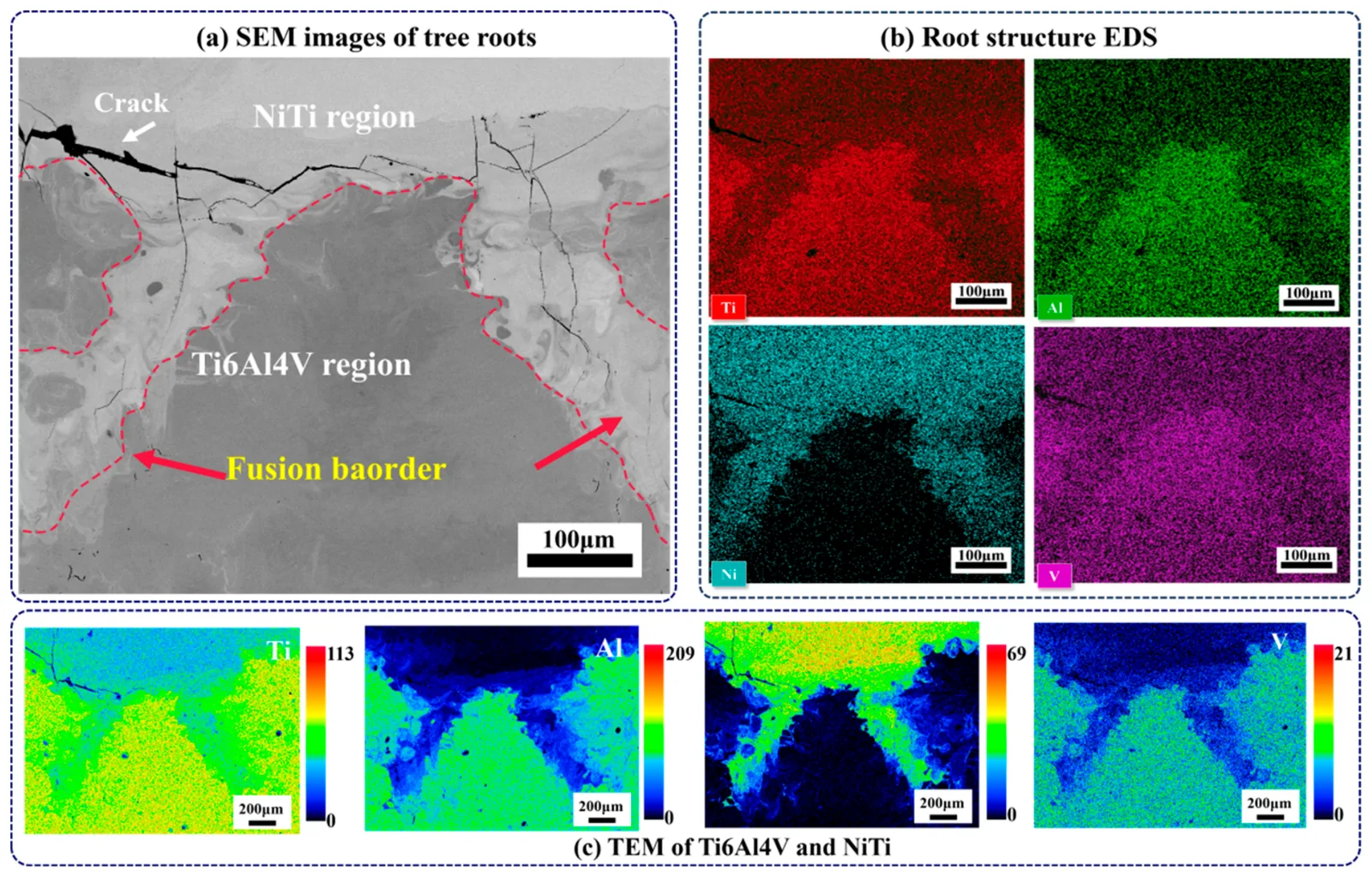
Elemental distribution characteristics at the interface of the bio-inspired joint: (**a**) SEM morphology; (**b**) EDS elemental mapping; (**c**) EPMA quantitative distribution maps.

To further reveal the elemental diffusion mechanism of the root-inspired bio-structure during laser powder bed fusion, the 45–1.2 group (with a size multiplier of 1.2 for both the top diameter and branch length) is selected as the representative specimen for EDS and EPMA analysis in this section. Although this group exhibits certain local defects in macroscopic forming quality, its interfacial morphology is highly representative and clearly reflects the essence of the metallurgical reaction of dissimilar metals at the complex bio-inspired interface. Figure 7 shows the elemental distribution characteristics at the joint interface of the bio-inspired structure. EDS mapping (Figure 7b) reveals that Ni diffuses into the Ti6Al4V side and Al and V migrate into the NiTi side, indicating elemental interdiffusion and metallurgical reaction. EPMA quantitative mapping (Figure 7c) further reveals a gentle gradient of Ni concentration across the interface, indicating that the bio-inspired structure increases the local heat retention time, which is beneficial for sufficient interdiffusion of the elements.

**Figure 8:**
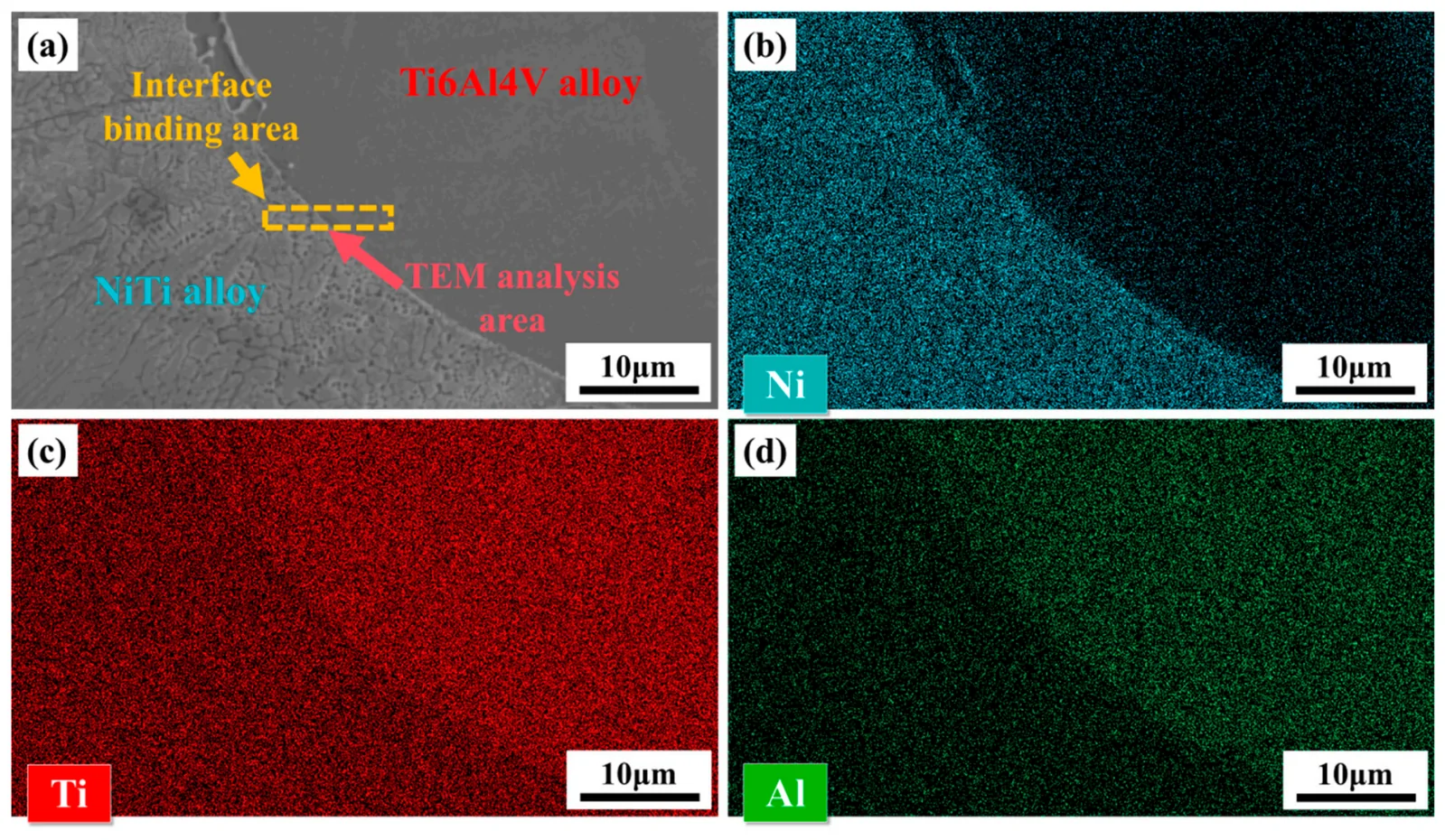
FIB sampling location and EDS elemental maps of the NiTi/Ti6Al4V interface: (**a**) SEM morphology of the FIB sampling area; (**b**) Ni; (**c**) Ti; (**d**) Al.

To reveal the nanoscale interfacial phases, a thin lamella was extracted from the typical fusion boundary of the 45–1.2 specimen via focused ion beam (FIB) milling and subsequently examined by transmission electron microscopy (TEM). Figure 8 presents the sampling location and the corresponding EDS elemental maps, which confirm bidirectional diffusion of Ni, Ti, and Al across the interface, forming a transition zone several micrometres in thickness.

The elemental interdiffusion confirmed by EDS and EPMA suggests the formation of interfacial reaction products. To identify these nanoscale phases and assess their role in the interfacial bonding, TEM and SAED analyses were conducted, as detailed in Section 3.3.

### 3.3. Interfacial Phase Analysis

**Figure 9:**
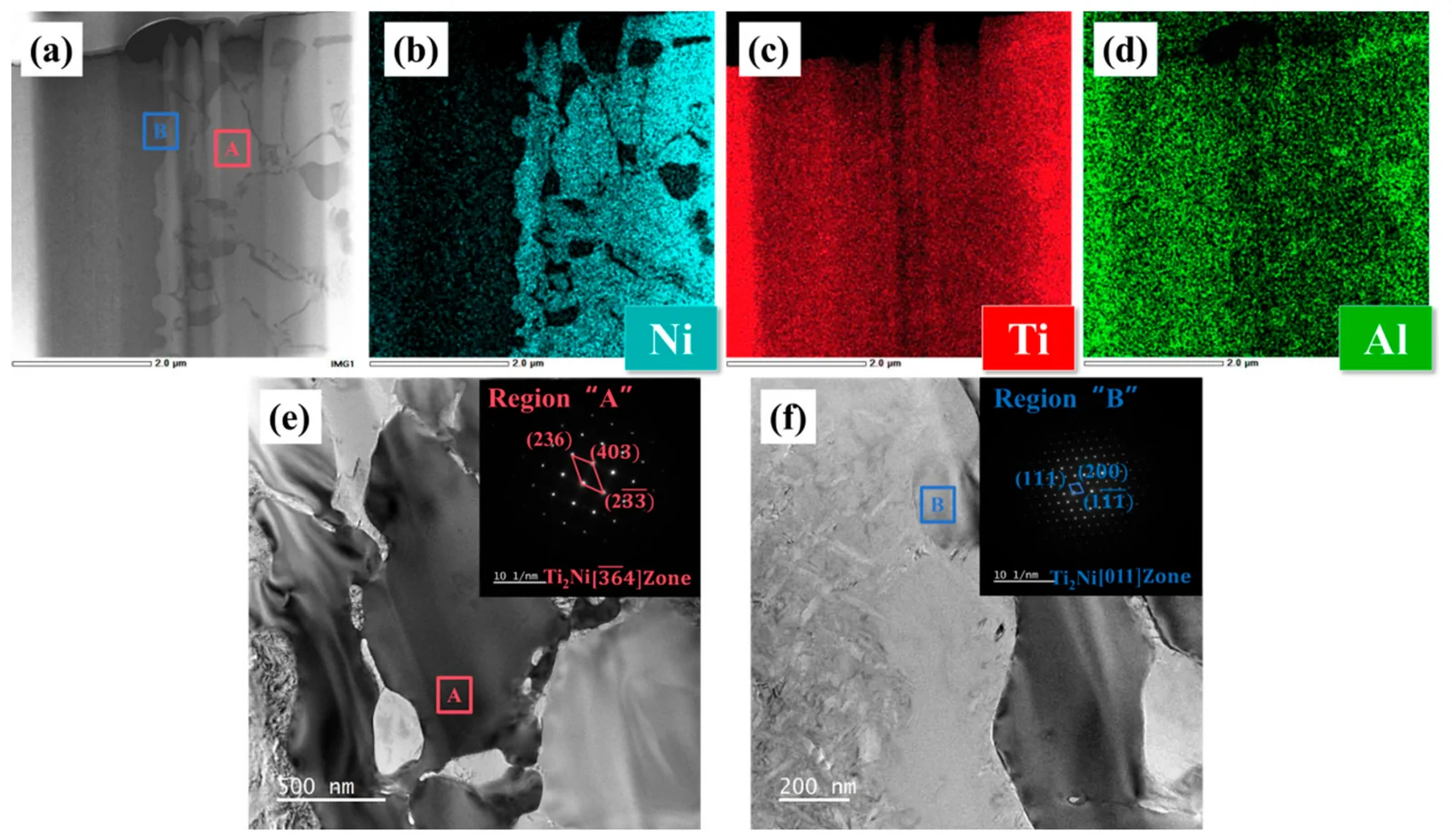
TEM elemental maps and diffraction pattern indexing of the NiTi/Ti6Al4V interface: (**a**) bright-field TEM image; (**b**–**d**) EDS maps of Ni, Ti and Al; (**e**) enlarged image of Region A with diffraction pattern; (**f**) enlarged image of Region B with diffraction pattern.

Figure 9 presents the TEM analysis results. The panoramic bright-field image (Figure 9a) shows a solidified structure on the NiTi side and a dense structure on the Ti6Al4V side. EDS maps (Figure 9b–d) confirm element interdiffusion. SAED patterns (Figure 9e,f) indicate that the Ti_2_Ni intermetallic compound is formed both at the centre of the fusion zone (Region A) and at the Ti6Al4V diffusion front (Region B). This critical finding confirms that the root-inspired bio-structure does not eliminate the brittle phase at the microstructural level; therefore, its strengthening effect must originate from the forced intervention of the macroscopic geometry on crack propagation behaviour.

It should be noted that the NiTi powder used in this experiment has a Ni mass fraction of 54.5 wt.% (slightly higher than the equiatomic NiTi). According to the Ni-Ti binary phase diagram, this slightly Ni-rich composition favours the preferential nucleation and growth of the Ti_2_Ni phase under the non-equilibrium solidification conditions of laser processing. The EDS and TEM results confirm the existence of a continuous Ti_2_Ni reaction layer at the interface, which is highly consistent with the thermodynamic driving force originating from the original powder composition. This implies that to eliminate the brittle phase by metallurgical means, the original composition of the NiTi powder must be strictly controlled; in this study, through geometric structure design, performance improvement was achieved without changing this metallurgical defect.

### 3.4. Interfacial Strength Analysis

**Figure 10:**
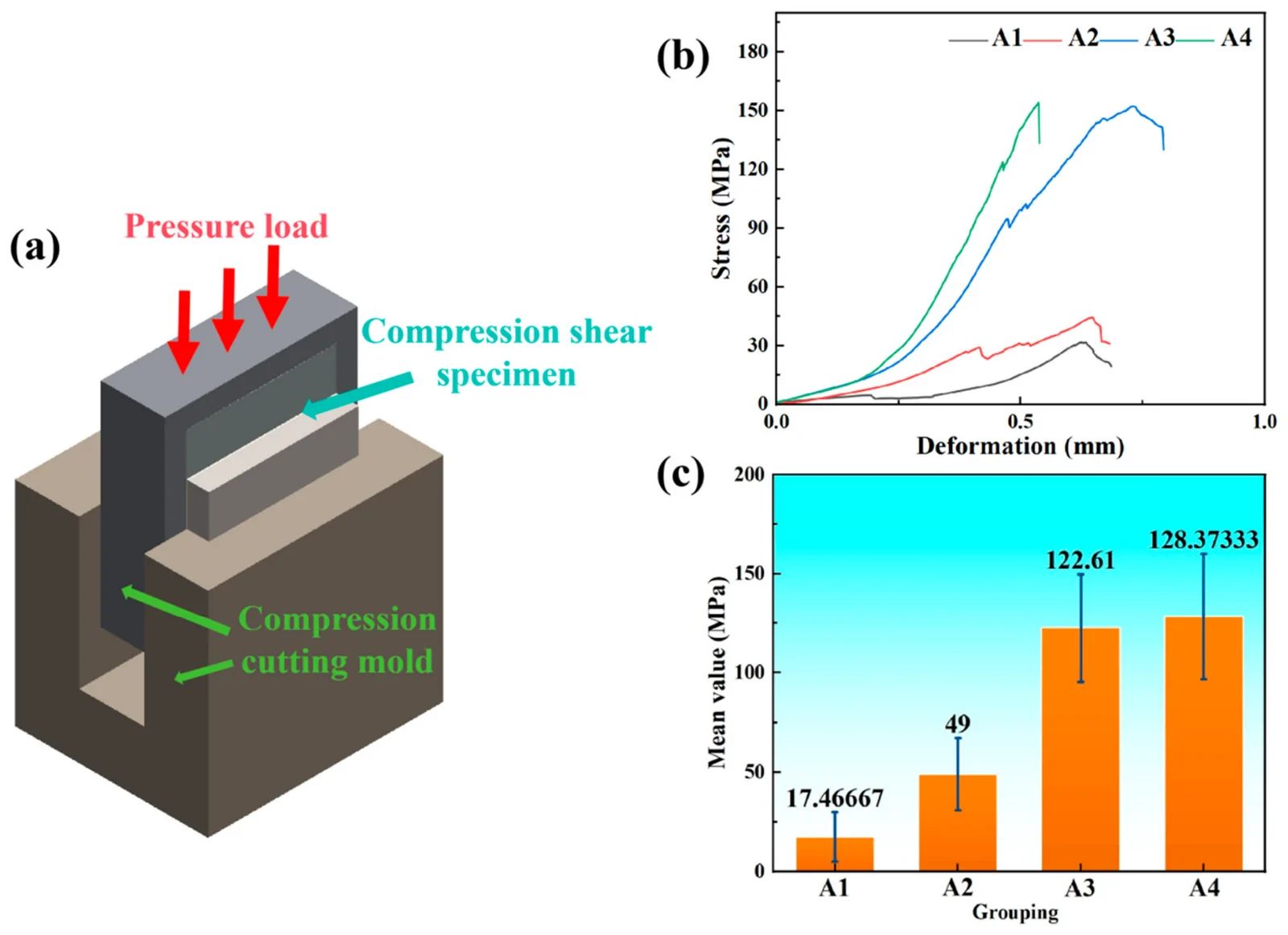
Compressive shear test results: (**a**) schematic of the compressive shear fixture and loading; (**b**) stress-displacement curves; (**c**) comparison of average shear strength.

Figure 10 shows the compressive shear test results. Figure 10a presents the fixture and loading schematic; Figure 10b shows the stress-displacement curves of the optimal specimens for the four groups; Figure 10c is a comparison of the average shear strength values.

The experimental results show that the branching angle is the primary factor determining the interfacial strength. The 45° branching groups (A1, A2) exhibit an average strength of only 17.47 MPa; in contrast, the 60° branching groups (A3, A4) show a dramatic increase to above 122.61 MPa. At the same branching angle, increasing the size multiplier from 1.2 to 1.5 provides additional strengthening but with a smaller contribution—A3 (60°, 1.2) reached 122.61 MPa, while A4 (60°, 1.5) reached 128.37 MPa. From the shape of the stress-displacement curves, the 45° groups exhibit a sudden drop after the peak, typical of brittle fracture; the 60° groups show higher shear stiffness and ductility characteristics, with a “step-like” phenomenon in the descending part of the curve, reflecting the progressive failure of the branch structures.

Combined with the quantitative crack density analysis in Section 3.1, the inferior shear strength of the 45° groups (only 17.47 MPa) can be largely attributed to their high crack area fractions (0.63% to 1.31%), where the extensive cracking drastically reduces the effective load-bearing cross-section of the interface, preventing efficient load transfer. In contrast, the 60° groups exhibit crack area fractions below 0.26%, maintaining much better structural integrity and allowing the geometric interlocking to fully exert its constraint-induced toughening effect, thereby activating mixed failure mechanisms such as crack deflection and branch shearing, ultimately leading to shear strengths exceeding 122 MPa. These results confirm a clear negative correlation between crack density and shear strength, and highlight that excessive cracking induced by low-angle branching is the critical factor responsible for strength degradation.

To establish a performance benchmark against the state-of-the-art, the shear strength obtained in this work is compared with that of conventional planar (two-dimensional) NiTi/Ti6Al4V interfaces reported in the recent literature. For instance, Bartolomeu et al. fabricated NiTi/Ti6Al4V multi-material interfaces via selective laser melting (SLM) without any geometric interlocking, and reported an average shear strength of only 33 MPa [20]. In sharp contrast, our optimised 60–1.5 bio-interlocking design achieves a shear strength of 128.37 MPa, which is approximately 3.9 times higher than that of the planar counterpart. This substantial improvement clearly demonstrates that the proposed macroscopic three-dimensional geometric interlocking strategy effectively compensates for the inherent metallurgical brittleness of the NiTi/Ti6Al4V interface—a level of enhancement that cannot be attained by conventional planar joints.

**Figure 11:**
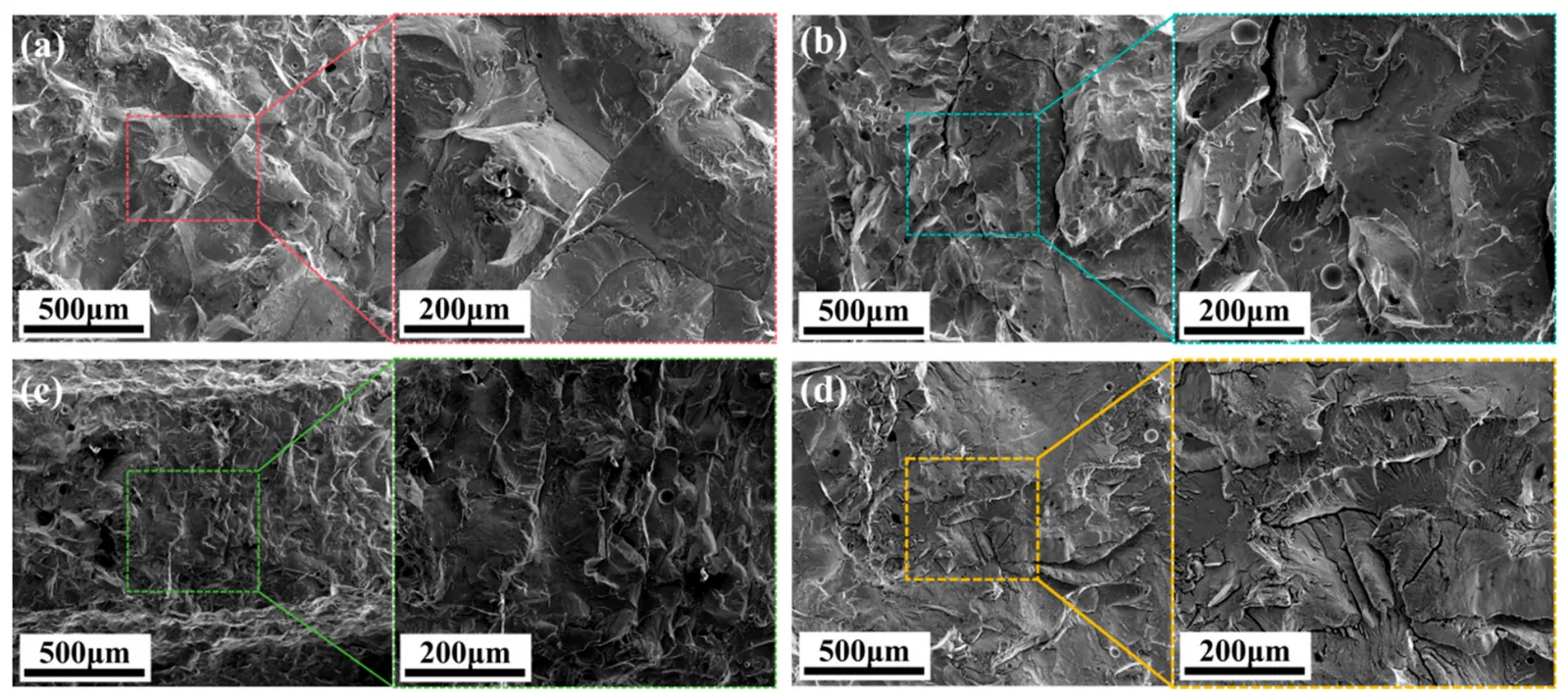
Interfacial fracture surface compositions of NiTi/Ti6Al4V with different design parameters: (**a**) 45–1.2 group; (**b**) 45–1.5 group; (**c**) 60–1.2 group; (**d**) 60–1.5 group.

Figure 11 shows the fractographic analysis. The 45° groups (Figure 11a,b) exhibit extremely flat fracture surfaces with large cleavage facets and river-like patterns, indicating typical brittle delamination along the interface. The 60° groups (Figure 11c,d) show distinct non-planar cracking features, with macro-wrinkles and crack deflection traces; the highest-strength A4 group (Figure 11d) also shows residual cavities and plastic deformation zones left after the forced shearing of the bio-branches.

**Figure 12:**
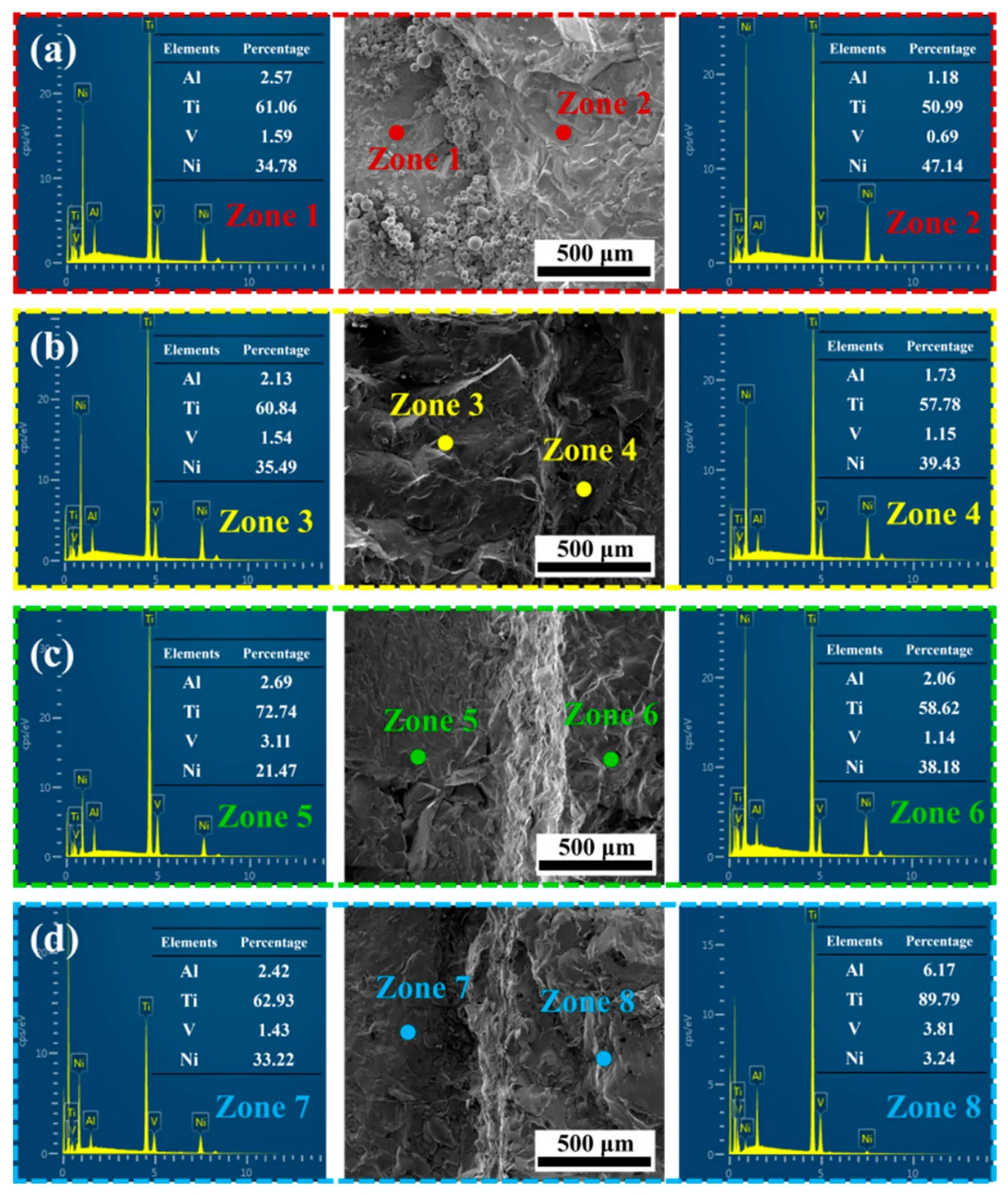
EDS point analysis of typical regions on the compressive shear fracture surfaces: (**a**) 45–1.2 group; (**b**) 45–1.5 group; (**c**) 60–1.2 group; (**d**) 60–1.5 group.

Figure 12 presents EDS point analysis of typical regions on the fracture surfaces. For the 45° groups (Figure 12a,b), the Ni atomic fractions on the fracture surface are 32.4–34.8% and Ti approximately 65%, which is highly consistent with the Ti_2_Ni phase composition, confirming that the crack propagated flatly along the continuous brittle reaction layer. For the 60° groups (Figure 12c,d), in addition to Ti_2_Ni, residues of Al and V are also detected—because V participates in the interfacial reaction to a very limited extent under the extremely high cooling rate of MM-LPBF, its presence confirms that the fracture path has partially moved away from the metallurgical reaction layer and penetrated into the Ti6Al4V base material, again corroborating the effectiveness of the crack-deflection toughening mechanism induced by geometric interlocking.

### 3.5. Comparison with Existing Interlayer-Based Methods

To contextualise the engineering significance of the proposed bio-interlocking strategy, Table 4 compares the present approach with representative interlayer-based methods for NiTi/Ti6Al4V joining reported in the literature.

While the Pd interlayer achieves the highest tensile strength, its prohibitive cost and complex processing window limit practical applications. The Cu and Nb interlayers offer moderate performance but still rely on precise thickness control and remain susceptible to brittle phase formation. In contrast, the proposed bio-interlocking design achieves competitive shear strength (128.37 MPa) without any intermediate material, significantly reducing both material cost and process complexity. Moreover, the geometric parameters (branching angle, size multiplier) can be readily adjusted to accommodate different material systems and loading conditions, offering substantially greater process flexibility.

In addition to interlayer-based methods, other bio-inspired interfacial architectures reported in additive manufacturing also merit comparison. Tree-like branching structures are primarily designed for load distribution but may lack the multi-axial constraint required for interfacial crack suppression. Tooth-like or jigsaw-interlocking sutures offer enhanced flexural resistance but are often limited to two-dimensional geometries. Hook-and-loop inspired designs enable reversible interlocking but are less effective under monotonic shear loading. Nacre-inspired brick-and-mortar architectures provide toughness enhancement through controlled crack deflection, yet their fabrication typically requires layer-by-layer assembly, which is less compatible with the single-step MM-LPBF process. In contrast, the present root-inspired design integrates the advantages of three-dimensional constraint, geometric tunability, and process compatibility, making it particularly suitable for MM-LPBF of dissimilar metals.

### 3.6. Practical Considerations for Industrial MM-LPBF Implementation

The translation of the root-inspired bio-interlocking design from laboratory-scale demonstration to industrial MM-LPBF production requires careful consideration of several practical factors.

Printing accuracy: The minimum feature size of the root structure (sphere diameter of 0.2 mm) is well within the resolution capability of current commercial LPBF systems (typical laser spot size ~50–100 μm). However, the dimensional accuracy of the branch tips may be affected by powder spreading inconsistencies and thermal distortion during layer-wise fabrication.

Build time: The additional time required for selective powder deposition and the fabrication of complex root geometries is estimated to increase build time by approximately 15–25% compared to planar interfaces, depending on the structural density and branching complexity.

Scalability: The current design has been validated at the lab scale (specimen dimensions of 8.7–12.6 mm). For larger components, the root structure dimensions may need to be scaled proportionally to maintain effective load transfer, while the increased thermal gradients in larger parts may necessitate adaptive scanning strategies to mitigate distortion.

Dimensional tolerances: Post-build inspection of the root-inspired structures using micro-CT or optical metrology is recommended to verify that the as-built geometry conforms to design specifications. The current results suggest that the 60° branching configuration is more tolerant to dimensional variations than the 45° configuration, as evidenced by its superior mechanical performance despite similar levels of geometric complexity.

## 4. Conclusions

This study addresses the critical issue of easy interfacial cracking in MM-LPBF fabricated NiTi/Ti6Al4V dissimilar metal components caused by the continuous Ti_2_Ni brittle phase. A root-inspired three-dimensional bio-interlocking interface structure is innovatively proposed, and the effects of branching angle and size multiplier on interfacial strengthening are systematically investigated without relying on any intermediate interlayer materials. The main conclusions are as follows:

(1) The effectiveness of the “geometrical constraint toughening” strategy is proposed and validated. Although TEM confirms that a continuous Ti_2_Ni brittle phase still exists at the interface, the optimised 60–1.5 structure increases the average shear strength to 128.37 MPa, which is more than six times higher than that of the conventional 45–1.2 group (17.47 MPa), fully demonstrating that macroscopic geometric design can effectively compensate for metallurgical brittleness.

(2) The dominant role of the branching angle and the toughening mechanism is revealed. For the 45° low-angle branches, owing to insufficient normal embedding depth, cracks propagate rapidly along the flat metallurgical reaction interface, and the fracture surface exhibits typical brittle cleavage characteristics with a composition highly consistent with the Ti_2_Ni phase. For the 60° high-angle branches, the increased normal embedding depth forces crack deflection and penetration into the Ti6Al4V matrix, resulting in a fracture surface showing non-planar wrinkles, residual cavities from sheared branches, and plastic tearing of the matrix. The failure mode changes from interfacial brittle delamination to a mixed progressive failure mode.

(3) Quantitative fracture characterisation provides direct evidence for the proposed toughening mechanism. ImageJ-based measurements of crack tortuosity and deflection angle reveal that the 60–1.5 group exhibits the highest tortuosity (T = 1.28) and the most substantial deflection angle (45–55°), confirming that the bio-interlocking geometry successfully forces the crack to deviate significantly from the flat metallurgical reaction layer, consuming substantially more fracture energy. These quantitative data are fully consistent with the observed shear strength trend.

(4) A systematic comparison with existing interlayer-based methods demonstrates the engineering advantages of the proposed strategy. While Pd interlayers achieve higher tensile strength, their prohibitive cost and complex processing windows limit practical applications; Cu and Nb interlayers offer moderate performance but remain susceptible to brittle phase formation. In contrast, the bio-interlocking design achieves competitive shear strength (128.37 MPa) without any intermediate material, significantly reducing both material cost and process complexity while offering substantially greater process flexibility through tunable geometric parameters.

In summary, this work establishes a new paradigm of “geometry instead of metallurgy” for dissimilar metal toughening. This strategy, relying solely on macroscopic interface structure design, enables load redistribution from the brittle reaction layer to the bulk matrix. It provides important theoretical guidance and engineering value for multi-material additive manufacturing of a wide range of other difficult-to-weld dissimilar metal combinations such as copper/steel and titanium/steel.

The present study primarily focuses on the macroscopic geometrical effects of the bio-interlocking design, while the potential influence of geometrical parameter variations on solidification behaviour, thermal gradients, and grain structure has not been systematically investigated. A comprehensive analysis of grain size and crystallographic texture across different branching angles and size multipliers would require extensive additional EBSD characterisation, which is beyond the scope of the current study. This limitation is recognised, and detailed correlation between the root-inspired geometrical parameters and microstructural evolution will be pursued in future work.

## Figures and Tables

**Table 1:** Chemical composition of NiTi, Ti6Al4V, and Nb powders (wt.%).

	Ni	Ti	Nb	Ta	Al	O	C	Fe	N	Mo	Cr	V
NiTi	54.5	Bal.	-	-	-	0.07	0.03	0.01	-	-	-	-
Ti6Al4V	-	Bal.	-	-	5.5	0.2	0.08	0.03	0.05	-	-	3.5
Nb	0.01	-	Bal.	0.03	-	0.035	-	0.03	-	0.01	0.05	-

**Table 2:** Experimental parameter matrix of the root-inspired bio-structure.

Specimen ID	Branching Angle θ	Size Multiplier	Shear Strength (MPa)
A1	45°	1.2	17.47 ± 1.81
A2	45°	1.5	-
A3	60°	1.2	122.61 ± 10.54
A4	60°	1.5	128.37 ± 15.03

**Table 3:** Quantitative crack propagation parameters measured from interfacial SEM images.

Specimen ID	Projected Length Lp (μm)	Actual Path La (μm)	Tortuosity T	Max Deflection Angle θd, Max
A1 (45–1.2)	~410	~460	1.12	~20°
A2 (45–1.5)	~460	~560	1.22	~30°
A3 (60–1.2)	~400	~440	1.10	~20–25°
A4 (60–1.5)	~400	~500	1.28	~45–55°

**Table 4:** Comparison of the proposed bio-interlocking design with existing interlayer-based methods for NiTi/Ti6Al4V joining.

Method	Interfacial Strength (MPa)	Fabrication Complexity	Process Flexibility	Manufacturing Cost
Cu interlayer (Oliveira et al.)	~300 (tensile)	Moderate	Limited	Moderate
Nb interlayer (Zhang et al.)	~90 (shear)	Moderate	Limited	High
Pd interlayer (Teshome et al.)	~520 (tensile)	High	Limited	Very high
This work (60–1.5 bio-interlocking)	128.37 (shear)	Low (no interlayer)	High (geometry tunable)	Low

## Data Availability

The original contributions presented in this study are included in the article. Further inquiries can be directed to the corresponding authors.
